# Land use intensity controls the diversity-productivity relationship in northern temperate grasslands of China

**DOI:** 10.3389/fpls.2023.1296544

**Published:** 2023-12-07

**Authors:** Yidan Yan, Lijun Xu, Xinjia Wu, Wei Xue, Yingying Nie, Liming Ye

**Affiliations:** ^1^ State Key Laboratory of Efficient Utilization of Arid and Semi-arid Arable Land in Northern China, Institute of Agricultural Resources and Regional Planning, Chinese Academy of Agricultural Sciences, Beijing, China; ^2^ Department of Geology, Ghent University, Ghent, Belgium

**Keywords:** grassland productivity, species diversity, grazing, mowing, enclosure, climate

## Abstract

**Introduction:**

The diversity-productivity relationship is a central issue in maintaining the grassland ecosystem’s multifunctionality and supporting its sustainable management. Currently, the mainstream opinion on the diversity-productivity relationship recognizes that increases in species diversity promote ecosystem productivity.

**Methods:**

Here, we challenge this opinion by developing a generalized additive model-based framework to quantify the response rate of grassland productivity to plant species diversity using vegetation survey data we collected along a land-use intensity gradient in northern China.

**Results:**

Our results show that the grassland aboveground biomass responds significantly positively to the Shannon-Wiener diversity index at a rate of 46.8 g m^-2^ per unit increase of the Shannon-Wiener index in enclosure-managed grasslands, under the co-influence of climate and landscape factors. The aboveground biomass response rate stays positive at a magnitude of 47.1 g m^-2^ in forest understory grassland and 39.7 g m^-2^ in wetland grassland. Conversely, the response rate turns negative in heavily grazed grasslands at -55.8 g m^-2^, transiting via near-neutral rates of -7.0 and -7.3 g m^-2^ in mowing grassland and moderately grazed grassland, respectively.

**Discussion:**

These results suggest that the diversity-productivity relationship in temperate grasslands not only varies by magnitude but also switches directions under varying levels of land use intensity. This highlights the need to consider land use intensity as a more important ecological integrity indicator for future ecological conservation programs in temperate grasslands.

## 1 Introduction

The relationship between species diversity and ecosystem functions has been a fundamental subject in ecology, attracting continuous attention in the past few decades (Chapin et al., 2000; Hautier et al., 2015). Being one of the most important ecosystem properties, biodiversity provides key support to processes such as biomass accumulation, energy flow, and nutrient cycling in the ecosystems (Balvanera et al., 2006). In addition, changes in the diversity-productivity relationship have consistent consequences for the cycling of major elements on broad spatial scales, substantially affecting greenhouse gas fluxes between the biosphere and the atmosphere (Handa et al., 2014). Therefore, accurate insights into the diversity-productivity relationship are essential to enhance ecosystem resilience and mitigate climate change impacts on agriculture, forestry, and grassland systems (Isbell et al., 2015; Ye, 2023).

In the diversity-productivity relationship in plants, the mainstream opinion is inclined to a positive correlation which recognizes that increases in species diversity promote ecosystem productivity (Wang et al., 2019; Thakur et al., 2021), despite disputes on driving mechanisms. For example, in low-productive bioclimatic regions such as desert steppe, steppe, and semi-steppe rangelands, increases in species diversity are found to increase plant productivity (Omidipour et al., 2021) due to increased species asynchrony and the chance of compensatory dynamics (Isbell et al., 2015). Meta-analyses involving ecosystems in multi-regions also show that ecosystems become more stable with higher asynchrony as a result of higher species diversity (Lin et al., 2010). Even under circumstances where environmental covariates are considered, the relationship remains positive (Xu et al., 2021). Nevertheless, plenty of evidence exists to depict a more complex picture, where the diversity-productivity relationship can not only be positive but also negative and may follow a unimodal pattern, a U-shaped pattern, or no pattern at all (Levine et al., 2004; Duffy et al., 2017). As the multi-ecosystem meta-analysis of Hector and Bagchi (2007) reveals, while the plant productivity is positively correlated with the plant species diversity at the local scale, the diversity-productivity relationship at the regional scale, however, follows U- or reverse U-shaped patterns. The multiple interactions and feedback among biotic and abiotic factors are probably the most important drivers of such multi-faceted relationships between biodiversity and plant productivity (Tilman et al., 2012).

The relationships between plant productivity and the climatic and landscape factors are complex and multifaceted too. Overall, climatic factors such as air temperature and precipitation have direct influences on plant growth and productivity. Higher biomass accumulations are often observed in environments with higher precipitations and more suitable temperature ranges (Wu et al., 2021; Ye et al., 2023). In contrast, the effects of landscape factors such as elevation, slope, and landforms on vegetation biomass production are more difficult to observe, because these factors usually exert influences on plant productivity indirectly via, e.g., erosion and sedimentation processes (Thelemann et al., 2010) or interact with management practices to foster a beneficial vegetation mosaic (Xu et al., 2022a). In semiarid grasslands of northern China, for instance, the relatively stable levels of plant diversity and productivity in recent years are attributed to landscape-scale management measures of nitrogen addition and mowing (Wang et al., 2021). On the one hand, research findings suggest that suitable climate and landscape conditions facilitate aboveground biomass (AGB) accumulation (Chen et al., 2023). On the other hand, however, extensive climate-landscape interactions add complex, nonlinear patterns to the diversity-productivity relationship. As indicated by Broderick et al. (2022), plant productivity response to climate is constantly influenced by the legacy effects of climatic history on plant community, soil microbial activity, and nutrient cycling. Critically, the magnitude and direction of this legacy effect vary with the landscape, meaning that climate and landscape should always be considered together in plant productivity evaluations.

Livestock grazing is found to play an important role in the diversity-productivity relationship. In arid and semiarid grasslands, reduced plant species diversity associated with increasing grazing intensity might have strong negative impacts on both below- and aboveground productivities (Sanaei et al., 2023). Livestock grazing can influence plant and soil conditions through biomass consumption, trampling, and addition of nutrients in dung and urine (Xue et al., 2023), but the extent of the impacts depend upon grazing intensity and frequency, local climate, and the type of plant community (Bai et al., 2012). Grazing can also promote species composition and, according to Huston (1979), the highest plant species diversity might be found at intermediate grazing intensity. Additionally, grazing intensity is also linked to abiotic conditions and landscape contexts, because livestock tend to feed more in lowland than upland areas, meaning that livestock disturbances are less intense in higher elevations and/or higher slope positions (Chang et al., 2021).

Moreover, previous research in the region (Li et al., 2022) finds that mowing can trigger a direction change in the grassland diversity-productivity relationship. This finding was based on an in-situ, multi-year experiment in a single locality. Whether different land use types or intensities can trigger similar changes to the diversity-productivity relationship at larger scales is still an open question to debate. Here, based on land use intensity evaluation (Yan et al., 2021) involving regionally common grassland use types including wetland grassland, forest understory grassland, mowing grassland, grazing grassland, and degraded grassland under enclosure management, we aim to further explore the effects of land use type and intensity on the relationships between plant diversity and productivity. We hypothesize that at the landscape scales, the diversity-productivity relationship varies with the type and intensity of exogenous disturbances that the grassland ecosystem will receive. Specifically, the objectives of this paper are to: (1) develop a modeling framework that is capable of handling both the nonlinear effects of biotic/abiotic factors on grassland productivity and the interactions among these factors; (2) evaluate the diversity-productivity relationship in response to varying land use intensity (LUI) classes under the co-influence of climatic and landscape factors; and (3) identify priority areas for future research on the diversity-productivity relationship especially in temperate grasslands.

## 2 Materials and methods

### 2.1 Study area

**Figure 1:**
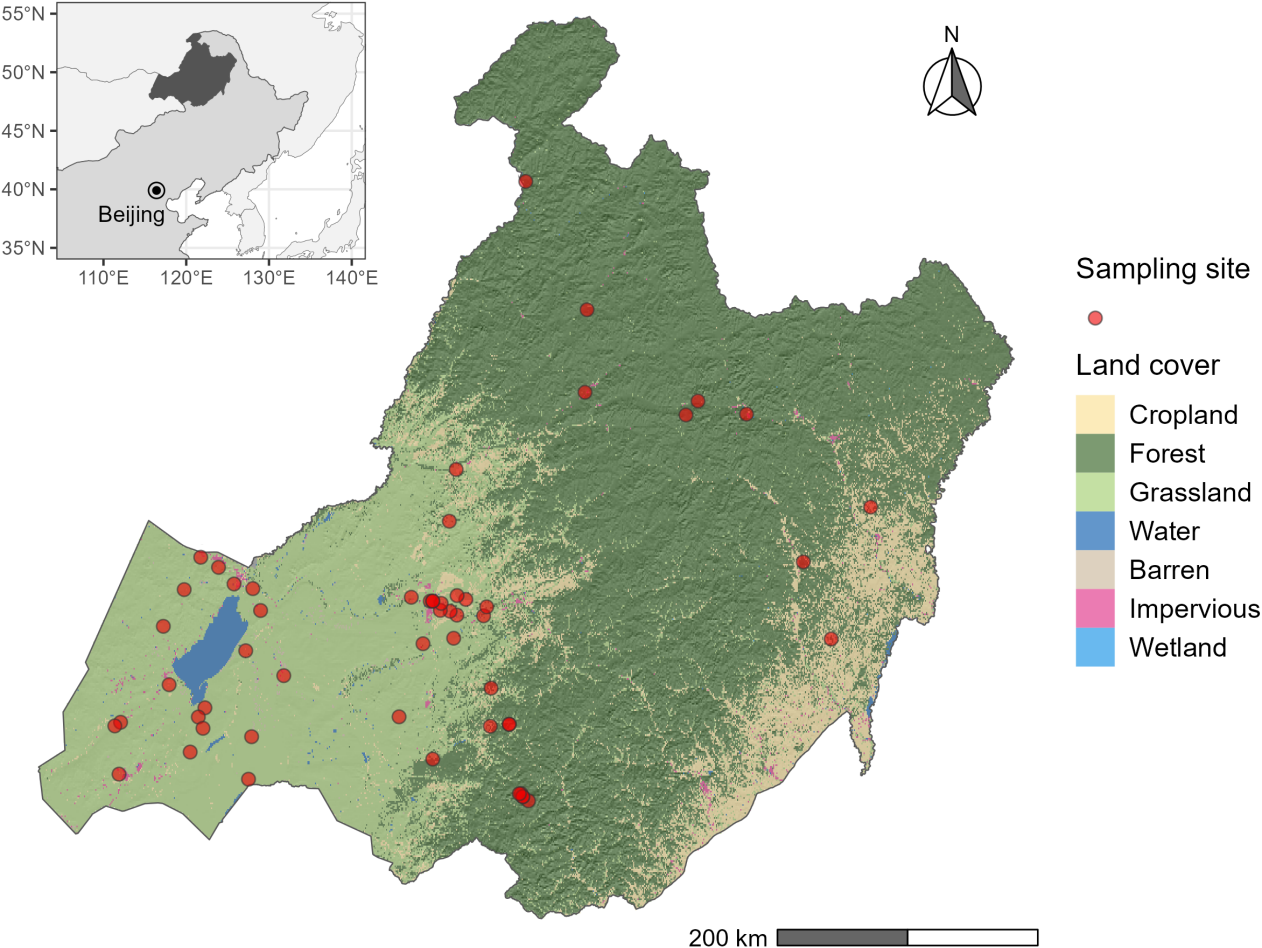
The study area. Plant sampling locations are indicated by the red dots. The underlying land cover types are extracted from the China Land Cover Dataset (Yang and Huang, 2021).

The research was conducted in Hulunber in northern Inner Mongolia, China (**Figure 1**). As part of the eastern Eurasian Steppe, Hulunber sits in the transition zone from the Greater Khingan Mountains to the Mongolian Highlands. A temperate continental climate prevails in the area. The annual precipitation totals 200-750 mm in the study area, 70% of which falls between June and August. The annual temperature averages between -4.5°C and 2.5°C. The regional vegetation possesses the mixed characteristics of the arid steppe, meadow steppe, and forest steppe. Dominant plant species are *Leymus chinensis* and *Stipa Baicalensis* in the area, where a range of associated species including *Carex spp*, *Cleistogenes squarrosa*, *Poa sphondylodes*, *Achnatherum sibiricum*, etc., coexist. The major soils in the study area are Kastanozems, Solonchaks, and Gleysols (IUSS Working Group WRB, 2015).

### 2.2 Vegetation survey

We conducted a vegetation survey in 55 sampling sites across the whole study area in July and August 2022 (**Figure 1**). These sampling sites cover all types of temperate grassland in the region, including grazing grassland, mowing grassland, degraded grassland under restoration (or enclosed grassland), wetland grassland, and forest understory grassland. The stocking rate in the heavily grazing grassland was evaluated as 0.92 AU ha^-1^, where 1 AU is defined as 500 kg cattle. In mowing grassland, hay was harvested by mowing annually in autumn. The restoration grassland was fence-enclosed year-round. Regional research (Yan et al., 2021) showed that the LUI in these grasslands is ranked in increasing order as: Enclosed grassland< forest understory grassland< wetland grassland< mowing grassland< moderate-grazing grassland< heavy-grazing grassland. At the time of sampling, no grassland was classified as the light-grazing class.

In each sampling site, three quadrats of 50 cm x 50 cm in size were randomly placed in vegetation, and plant communities within each quadrat were surveyed to determine the coverage, height, density, and AGB for each plant species that appeared within the quadrat. Considering that shrubs are rare in grassland ecosystems, we only considered quadrats without shrubs as they are more representative of grassland productivity. The area under vegetation cover and the area of bare soil were visually estimated *in situ* by experienced field staff and the percentage of vegetative area was derived as the coverage per plant species. Plant height was determined by the average height of three randomly selected plant individuals per species identified in situ. Plant density was measured by counting the total number of plant individuals per species. AGB was determined by collecting and weighing the aboveground part of the plant per species in each quadrat. Standing plants were cut at the soil surface per species and collected in sample bags. Fallen, withered parts on the soil surface were also collected. Dry weights were measured in the laboratory after the samples were oven-dried at 85°C for 12 h. The geographic coordinates and landscape vegetation types were simultaneously measured and described for each sampling site.

### 2.3 Climatic and landscape parameters

We obtained the fifth-generation ECMWF atmospheric reanalysis of land monthly averaged data (ERA5) from the Copernicus Climate Change Service (https://cds.climate.copernicus.eu/; accessed 1 March 2022). ERA5 is a state-of-the-art global reanalysis dataset for land applications, which provides a consistent spatiotemporal representation of the global climate system (Muñoz-Sabater et al., 2021). The spatial resolution of ERA5 is about 5 km in the study area. We derived mean annual temperature (MAT), mean annual precipitation (MAP), and mean annual potential evapotranspiration (PET) from monthly values in the sampling year and during the last ten years (MAT10, MST10, MAP10, and PET10, respectively) in trying to capture the effects of annual climate variability versus decadal climate trends on plant productivity (Cuo et al., 2021). The aridity index, which is defined as the MAP to PET ratio (UNEP, 1992), during the sampling year and the last ten years (AI and AI10, respectively) was also derived. The elevation data was obtained from the Shuttle Radar Topography Mission (Farr et al., 2007). The slope gradient and direction (i.e., aspect) were derived from the elevation data using the *terra* package in R (Hijmans, 2023).

### 2.4 Diversity index

We used the Shannon-Weiner diversity index (SnW) to measure the species diversity of the surveyed plant communities. SnW is defined by Equations 1 and 2 (Spellerberg and Fedor, 2003):


(Eq. 1) $$SnW=-\sum \left(P_{i}\cdot lnP_{i}\right)$$

(Eq. 2) $$P_{i}=\frac{1}{4}\left(RC+RH+RD+RB_{a}\right)$$

where *RC* is the relative coverage of a plant species; *RH* is the relative height; *RD* is the relative density; and *RB_a_* is the relative AGB. Here, the values of *RC*, *RH*, *RD*, and *RB_a_* are defined as the percentage of a plant species’ coverage, height, density, and AGB to the sum of all plant species identified in the plant community, respectively. It is important to note that the Shannon-Wiener index is a comprehensive representation of the plant species diversity, which simultaneously considers a plant species’ distribution extensiveness, competence, abundance, and productivity using *RC*, *RH*, *RD*, and *RB_a_*, respectively. In addition, the Shannon-Wiener index is sensitive to the changes in the coverage, height, density, or biomass of a species in the plant community. Since coverage, density, and biomass are relatively independent aspects of the plant community, the Shannon-Wiener index is less prone to the disturbance of statistical outliers (Leinster and Cobbold, 2012).

### 2.5 Model development

We developed a multiple regression model between AGB and plant diversity. In addition to the Shannon-Wiener index, sampling site-specific climatic and landscape factors, and LUI were also included as regressors. The model has the following form:


(Eq. 3) $$AGB=\alpha +\beta_{1}\cdot SnW+\beta_{2}\cdot s\left(CL\right)+\beta_{3}\cdot s\left(LS\right)+\beta_{4}\cdot SnW\times LUI+\varepsilon$$

where *CL* is a combination of MAT, MAP, PET, and AI, *LS* is a combination of elevation, slope gradient, and aspect; *s* is a smooth function used to capture the nonlinear relationships between a regressor and AGB. Cubic splines are a typical form of the *s* function (Wood, 2003); and symbol × stands for the interaction between two regressors, as in *SnW* × *LUI* for the interaction between the Shannon-Wiener index and LUI; ε is the model residual. It is important to note that *LUI* is a categorical variable, whereas all the other regressors are continuous variables. It is also important to note that the inclusion of an interaction term between *SnW* and *LUI* will produce a varying coefficient for *SnW* per LUI class, which makes the modeling of a varying AGB response rate to the diversity index per LUI class possible. The model was estimated in R (version 4.1.3) using the maximum likelihood method as implemented by the generalized additive model (GAM) function of the *mgcv* package (Wood, 2011). The spatial autocorrelation in ϵ was evaluated using the Moran’s I index as implemented in the *spdep* package (Bivand and Wong, 2018).

We constructed two contrasting models (i.e., LM versus GAM) to characterize AGB’s response to the three categories of covariates, namely, plant diversity, climate, and the plant community’s landscape position, conditioned by the LUI classes that represent the anthropogenic disturbances to the grassland ecosystem. A fundamental difference between these two models is that the interaction between plant diversity and LUI is included as an independent regression term in the GAM model, but not in the LM model because the term is tested insignificant in the latter. Only the most significant covariate per covariate category is retained in the models. To represent the nonlinear responses of AGB to plant diversity and the environment, the LM model adopts polynomial functions for the Shannon-Wiener index and MAT, respectively, whereas the GAM model employs a cubic spine function. Since LUI is a categorical variable, it is treated as fixed effects during model estimation (Lee et al., 2010).

We evaluated the performance of the candidate regression models using the metrics of the mean absolute error (MAE), the mean absolute percentage error (MAPE), the root mean square error (RMSE), and the coefficient of determination (R^2^). We also assessed the relative importance of the model regressors in terms of their R^2^ contributions averaged over the orderings among regressors (Lindeman et al., 1980), using the *relaimpo* package in R (Grömping, 2006). To increase the robustness of the relative importance assessment, we employed the bootstrap technique for deriving the mean and 95% confidence interval of the relative importance percentage for each model regressor based on 1,000 replications (Dikta and Scheer, 2021).

## 3 Results

### 3.1 Plant productivity and diversity

**Figure 2:**
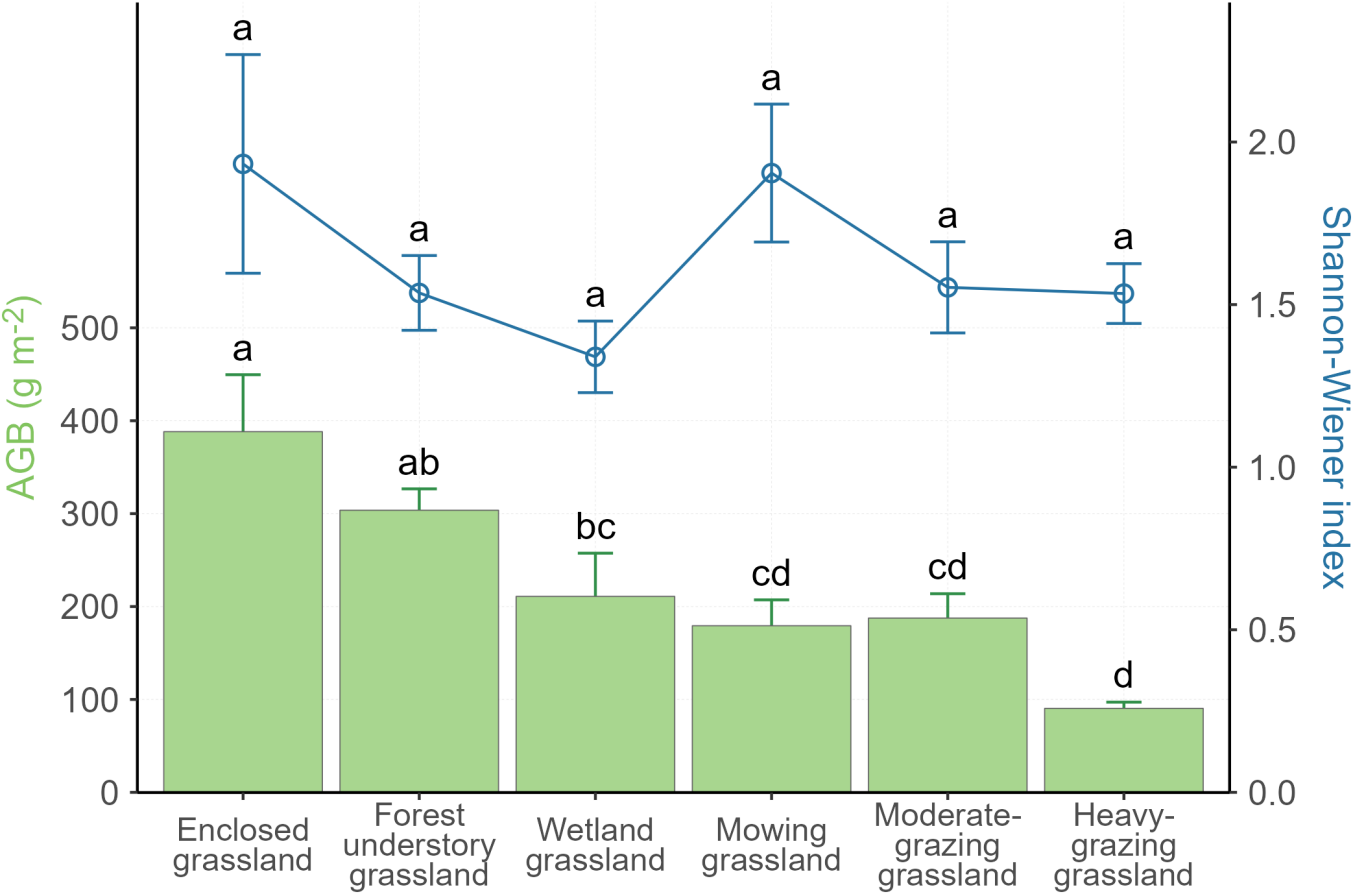
Variations in plant aboveground biomass (AGB) and the Shannon-Wiener index per land use intensity class. Error bars are standard errors. Different letters indicate significant differences at *P*< 0.05.

Among the 55 sites we sampled (**Figure 1**), plant AGB, MAT, and slope gradient vary among LUI classes. The survey results (**Table 1**) show that AGB varies greatly across the study area with a wide range between 32.6 g m^-2^ and 510.5 g m^-2^. The lowest AGB is observed in the heavily grazed grassland, while the highest AGB is observed in the grassland under enclosure management (hereafter enclosed grassland). The lowest MAT of -0.73°C is observed in forest understory grassland and the highest MAT of 1.68°C in the heavily grazed grassland. It is worth noting that the forest understory grassland is located at the highest elevation (846.09 m), whereas the heavy-grazing grassland is located at much lower elevations (600.89 m), only higher than the wetland grasslands (586.11 m). Although the Shannon-Wiener diversity index varies from 1.34 (wetland grassland) to 1.93 (enclosed grassland) in the study area, the differences in the Shannon-Wiener index among the LUI classes are insignificant (**Figure 2**).

**Table 1:** Plant aboveground biomass (AGB), Shannon-Wiener index, mean annual temperature (MAT), and slope gradient (%) per land use intensity (LUI) class in Hulunber.

LUI class	Samples	AGB	Shannon	MAT	Slope
		(g m^-2^)		(°C)	(%)
Enclosed grassland	3	388.23 ± 61.32	1.93 ± 0.34	0.34 ± 0.11	2.73 ± 0.73
Forest understory grassland	15	303.68 ± 22.99	1.54 ± 0.11	-0.73 ± 0.33	7.30 ± 1.23
Wetland grassland	6	210.91 ± 46.52	1.34 ± 0.11	0.16 ± 0.66	1.13 ± 0.21
Mowing grassland	7	179.32 ± 27.88	1.90 ± 0.21	1.01 ± 0.36	1.82 ± 0.61
Moderate-grazing grassland	5	187.58 ± 26.22	1.55 ± 0.14	0.85 ± 0.57	2.03 ± 0.78
Heavy-grazing grassland	19	90.38 ± 6.72	1.53 ± 0.09	1.68 ± 0.26	1.10 ± 0.20
Average	N = 55	198.10 ± 16.00	1.58 ± 0.06	0.62 ± 0.20	3.06 ± 0.50

Values are given as mean ± standard error.

### 3.2 Correlations between AGB and covariates

**Figure 3:**
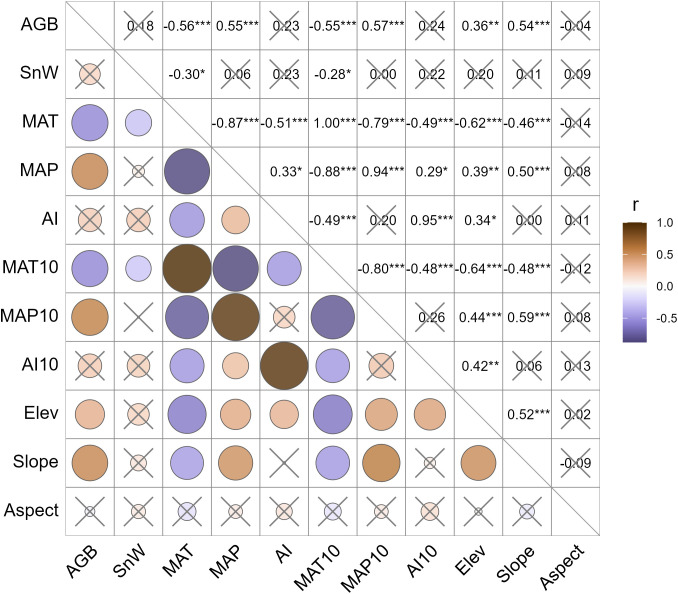
Pearson correlation coefficients between the cross pairs of the plant productivity, diversity, climatic, and landscape factors. The sign and magnitude of the correlation coefficient, *r*, are represented by the color and size of the circles. Statistically insignificant correlations are crossed out. AGB, aboveground biomass (g m^-2^); SnW, Shannon-Wiener index (dimensionless); MAT, mean annual temperature (°C); MAP, mean annual precipitation (mm); AI, aridity index (dimensionless); MAT10, MAP10, and AI10 are average MAT, MAP, and AI of the past 10 years, respectively; Elev, elevation (m A.S.L.); Slope, slope gradient (%); Aspect, slope direction. *, *P*< 0.05; **, *P*< 0.01; ***, *P*< 0.001.

AGB is significantly correlated with the selected climatic parameters (**Figure 3**). Specifically, AGB is positively associated with MAP, but negatively associated with MAT. We also considered MAT10 and MAP10 to examine whether the climatic legacy effect was valid on AGB. However, these two parameters are not significantly superior to their annual counterparts, MAT and MAP. Here, legacy climate in the past decade offers very limited extra power in explaining the AGB variabilities than the current climate. This is logical because grassland vegetation dynamics are predominantly coupled with the annual cycles. Decadal cycles are less relevant. Likewise, we tested the correlation between AGB and AI. To our surprise, the correlation coefficient between AGB and AI was not only much lower in magnitude than those for MAT and MAP but also insignificant. The correlation analysis confirmed our hypothesis that landscape factors are significantly positively correlated with the AGB, suggesting that plant communities tend to produce higher AGB at higher elevations or in steeper slopes. Last but not least, the Shannon-Wiener index was tested insignificant in association with the AGB across all LUI classes, although there was a tendency that AGB might increase by a small margin with higher plant diversity.

### 3.3 Established AGB models

**Figure 4:**
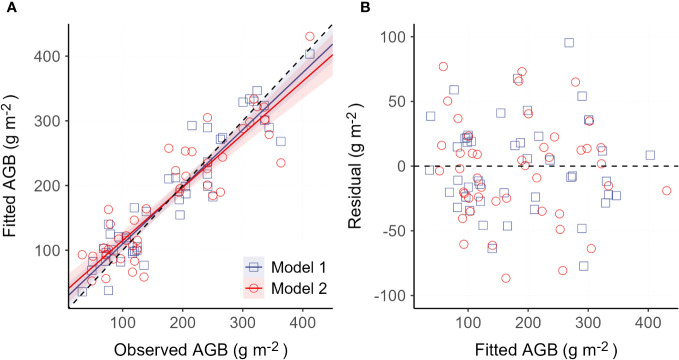
Validation of the two obtained AGB models. **(A)** Comparison between the field observed AGB and the model fitted AGB. Solid lines are linear trends for the two models, respectively. The dashed line is the 1:1 line. **(B)** Comparison between the model residual and model fitted AGB.

Established models (model 1 and model 2) are presented in **Table 2**. Model performance comparison shows that both model 1 and model 2 produce comparable fitting performance on our data (**Table 3**). It also shows that the model-fitted AGB values compare considerably well with the field-observed AGB and that no sensible patterns are discerned in the model residuals against the zero-error line for both models (**Figure 4**). This means that the variance of AGB is adequately characterized by both models and that additional covariates are not necessary. This also indicates that the detrimental effects of collinearity on the performance of the established models are negligible, despite correlations between, e.g., elevation and the climatic variables as shown in **Figure 3** (Hebbali, 2020). Spatial autocorrelation tests on the residuals of both models 1 and 2 show that the Moran’s I indices were evaluated as 1.03 for model 1 and 0.66 for model 2, suggesting that AGB residuals are slightly clustered among the vegetation sampling sites. The testing results also show that the *P*-values associated with the obtained Moran’s I indices are 0.28 and 0.48 for model 1 and model 2, respectively, meaning that extra statistical handling of the spatial autocorrelation in AGB residuals is unnecessary (Bivand and Wong, 2018). Although model 1 produces a slightly lower MAE (28.83 g m^-2^) than model 2 (32.56 g m^-2^), model 1 compares inferior to model 2 in terms of the other two metrics, MAPE and RMSE. Moreover, the goodness of fit measurement (R^2^) of model 1 is found lower than that of model 2. Critically, the Bayesian Information Criterion (BIC) value is evaluated at least 4 units higher for model 1 than for model 2, suggesting that model 2 is neater than model 1. Based on these comparisons, model 2 is adopted in the subsequent diversity-productivity relationship evaluation.

**Table 2:** Coefficients of the two obtained AGB models considering the Shannon index, mean annual temperature (MAT), slope gradient, and land use intensity (LUI) as regressors.

Variable	Category	Type	Coefficient	Std. Err.	*P*
Model 1					
Intercept	Intercept	Linear	-103.59	153.90	0.51
Shannon	Diversity	Polynomial			
		Order 1	920.62	316.20	0.006
		Order 2	-622.21	204.25	0.004
		Order 3	130.66	41.56	0.003
Slope	Landscape	Linear	11.00	3.04	<0.001
MAT	Climate	Polynomial			
		Order 1	-8.36	11.69	0.479
		Order 2	-16.24	5.41	0.005
		Order 3	5.28	2.61	0.051
LUI	Management	Categorical			
		Forest understory grassland	-56.26	41.84	0.188
		Wetland grassland	-23.17	43.40	0.597
		Mowing grassland	-157.48	34.82	<0.001
		Moderate grazing grassland	-128.15	38.11	0.002
		Heavy grazing grassland	-201.26	34.28	<0.001
Model 2					
Intercept	Intercept	Linear	167.24	34.70	<0.001
Shannon	Diversity	Linear	50.88	22.41	0.029
Slope	Landscape	Linear	12.00	3.49	0.001
MAT	Climate	Cubic spline			0.033
LUI	Management	Categorical			
		Forest understory grassland	-18.98	5.67	0.043
		Wetland grassland	14.41	5.35	0.058
		Mowing grassland	-53.97	17.59	0.004
		Moderate grazing grassland	-55.42	21.05	0.012
		Heavy grazing grassland	-102.48	17.34	<0.001

Nonlinear responses in AGB to Shannon index and/or MAT are considered using polynomial functions in model 1 and cubic spline functions in model 2. Model performance evaluation results are given in **Table 3**.

**Table 3:** Performance evaluation of obtained AGB models based on mean absolute error (MAE), mean absolute percentage error (MAPE), root mean square error (RMSE), determination coefficient (R^2^), and the Bayesian Information Criterion (BIC).

Model	MAE	MAPE	RMSE	R^2^	BIC
	(g m^-2^)	(%)	(g m^-2^)		
Model 1	28.83	25.93	42.37	0.81	528.42
Model 2	32.56	21.19	34.96	0.88	521.38

### 3.4 Relative importance

**Figure 5:**
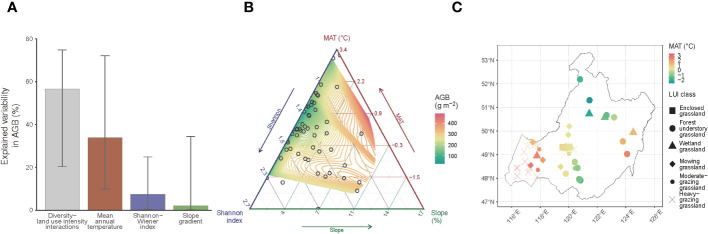
Driving factors of the grassland aboveground biomass (AGB). **(A)** Relative importance of Shannon-Wiener index, mean annual temperature (MAT), slope gradient, and the interactions between the Shannon-Wiener index and land use intensity (LUI) in explaining the AGB’s spatial variability. The filled bars represent the mean, and the whiskers represent the 95% confidence interval derived from the bootstrapping of 1,000 replications of the relative importance evaluation procedure (Grömping, 2006) using a generalized additive model (Equation 3); **(B)** AGB variations in response to the three most important factors identified in **(A)** namely, the Shannon-Wiener index, MAT, and slope gradient; **(C)** Spatial representation of the relationship between MAT and LUI classes per sampling site.

Based on the bootstrap evaluation of the regressor’s relative importance in explaining the spatial variability in AGB, we find that 33.9% of the AGB variability is attributed to MAT, relative to a much lower level of 7.4% for the Shannon-Wiener index (**Figure 5A**). The contribution of the plant community’s landscape position, represented by slope gradient, is somewhat marginal (2.1%). The primary control (56.6%) of the AGB variability comes from the interactions between plant diversity and LUI. The lower bounds of the 95% confidence intervals of the relative importance values are all higher than zero, suggesting that the mean importance values are all statistically significant (*P*< 0.05). We further examine the associated variations between AGB and its covariates using a ternary plot (**Figure 5B**). A general increasing trend of AGB with increasing slope gradient is evident in full ranges of MAT, covering all of the sampling sites under the observed LUI classes (**Figure 5C**). Although AGB displays a generally increasing trend with increasing MAT, the MAT-AGB relationship becomes less evident in the lower ranges of the slope gradient (slope< 4%), suggesting that herbivore removal of AGB are more intense in lowland areas. Likewise, a varying trend of AGB is observed in response to changes in the Shannon-Wiener index across the slope ranges of the landscape. While the AGB trend is more homogeneous against the Shannon-Wiener index in, e.g., forest understory grasslands (**Figure 5C**), the AGB trend is observed to vary more in mowing and heavily grazed grasslands.

In short, the varying trends in AGB in response to different categories of covariates suggest that the effects of LUI as the primary driver of the AGB variability have to be controlled in the first place if the diversity-productivity relationship were to be untangled.

### 3.5 Diversity-productivity relationship

**Figure 6:**
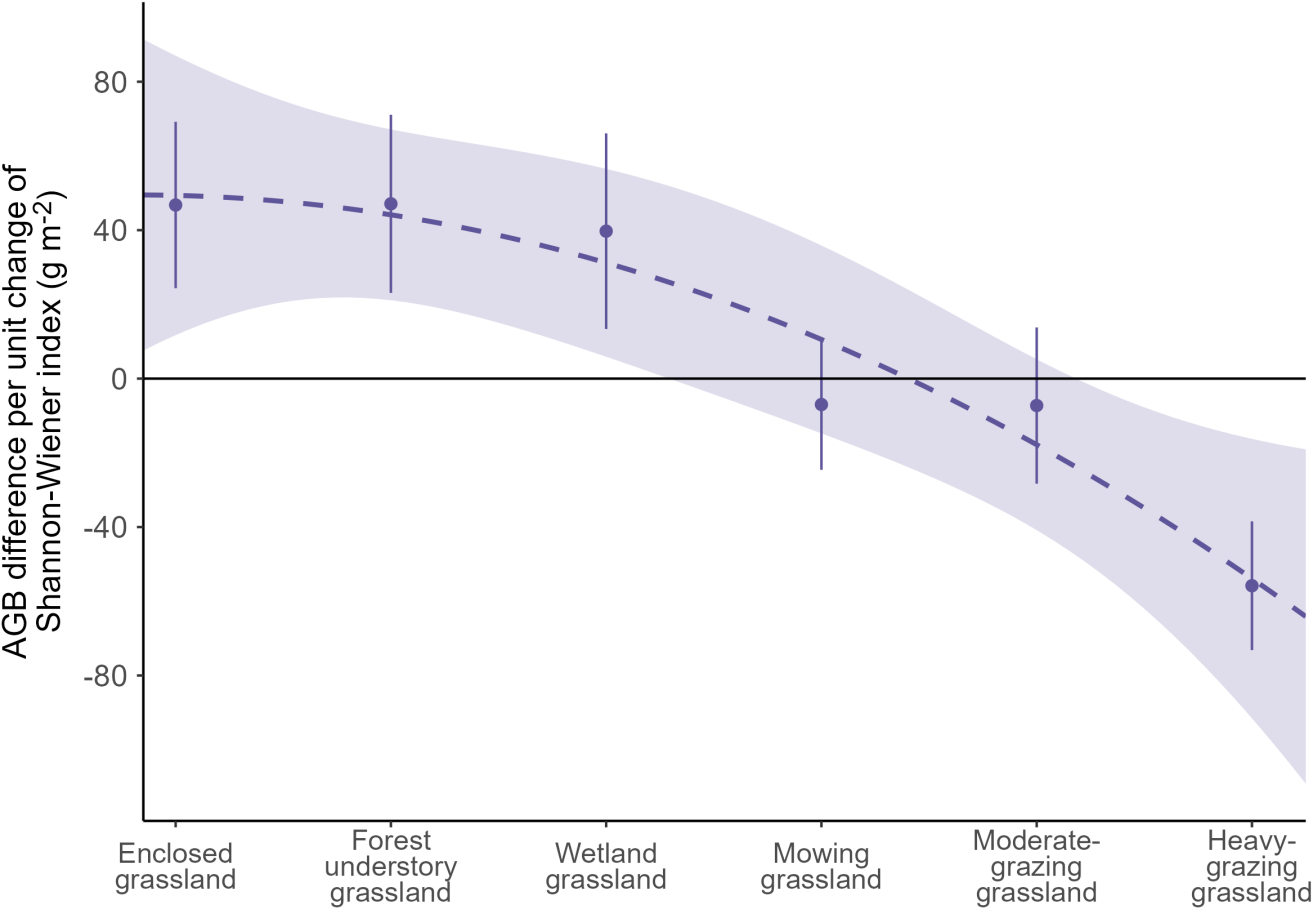
The average response rate of aboveground biomass (AGB) to the Shannon-Wiener diversity index taking all vegetation survey sites together per land use intensity class. The response rate is evaluated as the regression coefficient of the Shannon-Wiener index using a generalized additive model (Equation 3).

According to model 2, the AGB of the plant communities in enclosed grasslands is characterized to rise at a rate of 46.78 g m^-2^ per unit increase of the Shannon-Wiener index (**Figure 6**). The AGB response rate stays at comparably positive levels in forest understory grassland and wetland grassland (47.10 and 39.73 g m^-2^ per unit increase of Shannon-Wiener index, respectively), while in mowing grassland and moderate-grazing grassland, the AGB response rates decrease to -6.99 and -7.25 g m^-2^ for each unit increase of the diversity index, respectively. Conversely, for each unit increase of the Shannon-Wiener index, the model predicts that plant AGB in heavy-grazing grasslands decreases by 55.81 g m^-2^. This means that the response rate of the AGB to the Shannon-Wiener index drops from positive levels in the enclosed, forest understory, and wetland grasslands to a negative level in the heavy-grazing grasslands, transiting via near-zero levels in the mowing and the moderate-grazing grasslands.

## 4 Discussion

### 4.1 The modeling framework

With the model we developed and the dataset we collected, we find that grassland AGB responds significantly positively to plant species diversity in grasslands under enclosure management, the forest understory grassland, and the wetland grassland, contrasting to a potential negative response in AGB to the Shannon-Wiener diversity index in heavily grazed grasslands. We also find that the responses of grassland AGB to changes in plant diversity are neutral in mowing grassland and moderately grazed grassland. Moreover, we demonstrate that the diversity-productivity relationship exhibits a downward trend against the increasing LUI gradient as in the order of enclosed grassland, forest understory grassland, wetland grassland, mowing grassland, moderate-grazing grassland, and heavy-grazing grassland that we surveyed (**Figure 6**), meaning that the diversity-productivity relationship not only changes magnitude but also switches directions under varying levels of LUI. While these findings are in line with some authors for a more complex diversity-productivity relationship (Levine et al., 2004; Hector and Bagchi, 2007; Duffy et al., 2017), and confirm and extend previous research in the region (Li et al., 2022), these findings challenge the mainstream opinion that supports a consistently positive relationship between plant diversity and productivity (Isbell et al., 2015; Wang et al., 2019; Omidipour et al., 2021; Thakur et al., 2021).

Using a synthesis dataset of 854 grassland sites in Inner Mongolia, for example, Bai et al. (2007) tested the relationship between grassland productivity and species abundance in the whole of Inner Mongolia and found that the diversity-productivity relationship remained positive across multiple plant organizational levels and spatial scales. Bai et al. (2007) also found that the positive diversity-productivity relationship was robust against management practices such as mowing. In contrast, our results differ from both points. The diversity-productivity relationship that our results revealed varies from positive to negative under LUI levels from low (enclosed grassland) to high (heavy-grazing grassland), respectively, transiting via intermediate levels including the mowing grasslands in particular. Discrepancies in the dataset and model may be a major cause for this differential characterization of the diversity-productivity relationship. The dataset we use is produced by one survey team and in one field campaign, whereas the synthesis data used in, e.g., Bai et al. (2007) was produced by multiple teams and in at least multiple years, giving rise to data quality concerns (Fohrafellner et al., 2023).

Not only our dataset is unique, but our modeling framework is unique as well. We consider climatic and landscape factors in addition to plant diversity and anthropogenic disturbances in modeling grassland productivity using a framework that is capable of explicit handling of nonlinear effects and factor-to-factor interactions. In a similar effort to characterize the alpine grassland productivity in the Qinghai-Tibet Plateau, Wu et al. (2021) considered both climatic and anthropogenic contributions using spline functions in a GAM model structure. However, an explicit representation of the anthropogenic disturbance to the grassland ecosystem was missing from the approach. As a compromise, they interpreted the model residuals as the anthropogenic effect, in addition to the nonlinear effects of temperature, precipitation, and radiation. Likewise, in trying to model the nonlinear effects of multiple global change factors including temperature, precipitation, carbon dioxide concentrations, and nitrogen deposition on the net primary production of a California grassland, Zhu et al. (2016) simply employed quadratic functions. Additionally, although the role of interactions between biotic and abiotic factors has been recognized in eco-environmental research for a long time, proper handling of interactions in modeling approaches is still rarely seen (Duncan and Kefford, 2021). The modeling framework we develop here represents a significant endeavor in diversity-productivity relationship research, especially in the eastern Eurasian Steppe.

### 4.2 Diversity-productivity relationship

Our results support the consensus established in previous research that grassland productivity is simultaneously affected by multiple factors. These include, among others, plant diversity, climate, landscape, and management. The effect of plant diversity on productivity is currently disputed for magnitude, sign, and pattern (Hector and Bagchi, 2007; Duffy et al., 2017; Thakur et al., 2021), as already discussed above. Although our results suggest a potential positive effect of slope gradient on AGB, as further confirmed by its marginal importance of 2.1% in explaining the spatial variability in AGB, previous research showed that landscape position could have a stronger relationship (Wang et al., 2007) or a nonlinear relationship with the AGB (Sa et al., 2012). It is important to note that although slope gradient and AGB are positively correlated, the regression coefficient of it in a multiple regression model may stay positive or turn negative due to the suppressor effect (Watson et al., 2013). In this paper, MAT’s effect on AGB is well captured using either a polynomial or a spline function, exposing that grassland productivity responds nonlinearly to air temperature. This is largely in line with a range of previous research. For example, Lin et al. (2010) found that at the global scale, grassland AGB first increased to a peak level and decreased thereafter in response to moderate warming. A similar inverse U-shaped pattern was also found in AGB’s response to MAT in the Eurasian Steppe (Jiao et al., 2017). What was not frequently seen in previous research is that MAT can account for as high as one-third (33.9%) of the spatial variability in AGB, confirming the importance of air temperature in biomass accumulation (Anderson et al., 2006; Ye et al., 2013). Above all, our results reveal that the interactions between plant diversity and LUI account for over half (56.6%) of the variabilities in AGB, compared to 7.4% for plant diversity. The statistical establishment of this finding represents a major contribution of this paper to the diversity-productivity relationship research.

**Figure 7:**
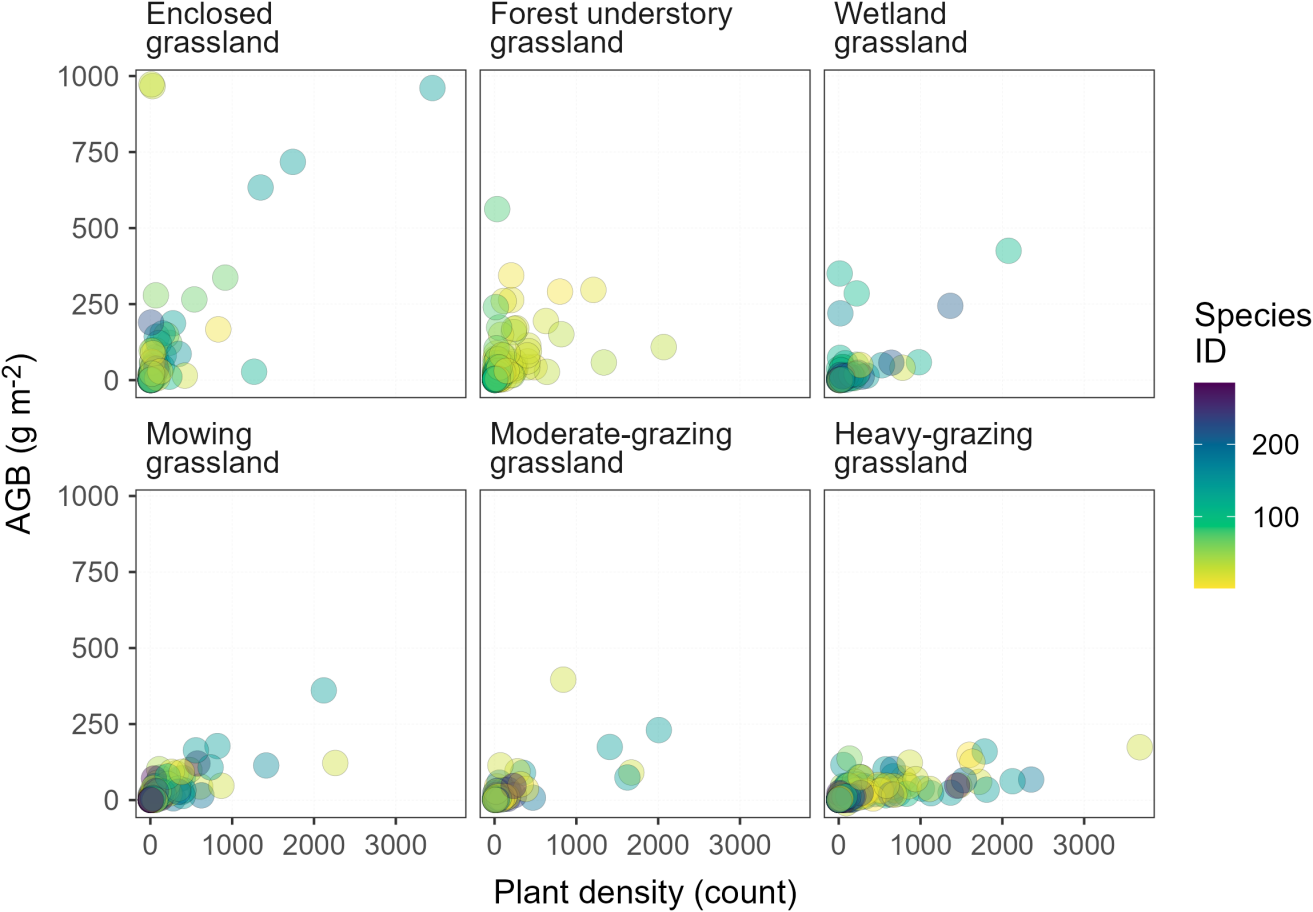
Relationship between aboveground biomass (AGB) and plant density in temperature grasslands. Rare species appear in the lower range of the x-axis, whereas non-rare species appear in the upper range of the x-axis in each panel.

Based on observational data on plant diversity and AGB, the diversity-productivity relationship characterized here may be fundamentally different from that obtained from field experiments. Most plant diversity experiments manipulate plant species by including the common, native species into the experimental design where rare and non-native species are usually unintentionally excluded (Dee et al., 2023). Rare and dominant species can affect productivity differently. An increase in species diversity resulting from dominant species usually increases productivity, however, increases in diversity that come from rare species decrease productivity (Parker et al., 2019). Among the plant species we observed through the vegetation survey, rare species are much more prevalent than the non-rare and, in particular, dominant species (Enquist et al., 2019) in terms of the total observed number of plant individuals per species across all LUI classes (**Figure 7**). The contrast between the rare and non-rare species is even higher in less-intensively managed grasslands, such as the enclosed grassland, forest understory grassland, and wetland grassland. One important observation in this study is that increases in LUI effectively eliminate the more productive species from the plant community in all grassland types (**Figure 7**). On the one hand, the rare species in more intensively managed grasslands may produce less AGB than dominant species (Parker et al., 2019). On the other hand, however, these rare species also compete with dominant species for space. Collectively, these productivity-reducing effects and the changing rare versus non-rare species composition drive the LUI-dependent diversity-productivity relationship in this paper.

### 4.3 Priorities for future research

We propose the following recommendations for future research. Firstly, diversity-productivity relationship research that involves multiple scales is much needed, and, therefore, should be encouraged. Research projects that unite observations and experiments from, e.g., the local (Wang et al., 2019), subregional (this paper), to regional scales (Bai et al., 2007) based on data integration and fusion should be prioritized. It is important, however, to ensure that the scale issues are correctly identified and handled (Duncan and Kefford, 2021), because the number of interactions and feedback among factors vary increasingly under multi-scale circumstances, driving greater complexity and uncertainty in diversity-productivity relationship characterization (Tilman et al., 2012). For example, climate variability and change usually show strong impacts on plant diversity and productivity at local scales. At regional scales, however, the impact may become more difficult to characterize due to confounding effects or shifting feedback mechanisms (Isbell et al., 2015). Secondly, multiple biotic and abiotic factors should be considered in modeling diversity-productivity relationships. We believe that relevant climatic and landscape factors should always be considered together, in addition to an appropriate proxy of anthropogenic disturbances. Although we opt for the LUI classes in this paper, other options – e.g., the Integrated Disturbance Index (Ligeiro et al., 2013) – need to be explored; Thirdly, the synergistic diversity-productivity relationship under moderate grazing or mowing management deserves more attention. On the one hand, there is a good chance that the dual goals of grassland utilization and conservation can be simultaneously met under these management schemes (Tälle et al., 2016; Li et al., 2022). On the other hand, although previous research suggested that diversity-productivity synergy could be fulfilled via, e.g., competition mediation (Peintinger and Bergamini, 2006; Fagundez, 2016) and plant-soil interactions (Storkey et al., 2015; Xu et al., 2022b), it is still unclear how this synergy can be reliably triggered and temporally sustained. More research is therefore needed.

## 5 Conclusions

Our research provides novel evidence that the grassland diversity-productivity relationship is controlled by LUI in temperate grasslands, based on the field data we collected and the modeling framework we developed for this purpose. Our major finding is that the response rate of grassland productivity to plant diversity decreases from positive to negative values along an increasing LUI gradient, which challenges the mainstream opinion that recognizes a robustly positive relationship between plant diversity and productivity. This highlights the need not only to prioritize the diversity-productivity relationship research, especially those involving multiple spatial scales, but also to incline to LUI as a viable indicator of ecological integrity especially for the temperate grassland ecosystems. This also indicates that there is a good chance to balance the use and conservation of grassland resources by adopting a moderate grazing or mowing scheme in grassland management. It is clear that heavy grazing should be avoided under all circumstances for the well-being of grassland ecosystems.

## Data availability statement

The original contributions presented in the study are included in the article/supplementary material. Further inquiries can be directed to the corresponding authors.

## Author contributions

YY: Data curation, Investigation, Software, Writing – original draft. LX: Conceptualization, Funding acquisition, Validation, Writing – review & editing. XW: Data curation, Investigation, Software, Writing – original draft. WX: Data curation, Investigation, Software, Writing – original draft. YN: Formal analysis, Project administration, Resources, Validation, Writing – original draft. LY: Conceptualization, Methodology, Supervision, Visualization, Writing – review & editing.
